# Spinal cord infarction after supraventricular tachycardia—A diagnosis not to be forgotten

**DOI:** 10.1097/j.pbj.0000000000000232

**Published:** 2023-10-16

**Authors:** Marta D. Martins, Ana Rocha, Marina Mendes, João Rocha, Inês Ferreira

**Affiliations:** a Internal Medicine Department, Centro Hospitalar Tâmega e Sousa, E.P.E. Penafiel, Portugal,; b Neurology Department, Centro Hospitalar Tâmega e Sousa, E.P.E., Penafiel, Portugal

## To the Editor:

Acute spontaneous spinal cord infarction (SCI) is rare, but can cause sudden onset of quadriplegia or quadriparesis.^1^ Some differential diagnoses which spinal cord infarction may mimic include cauda equina syndrome, space-occupying lesions, longitudinally extensive transverse myelitis, infection, and arteriovenous malformations compressing the spinal cord.^2^ The poor prognosis of spinal cord lesions is often due to treatment delay, and therefore, early diagnosis is essential.^3^ Magnetic resonance imaging (MRI) is a sensitive and reliable modality for the diagnosis of spinal cord infarction.^2^ Rehabilitation efforts have proven to be successful in achieving good outcomes.^4^ Control of risk factors is fundamental. The most frequent etiology (directly or through hemodynamical insufficiency) is atherosclerosis. Treatment with antiplatelet therapy is fundamental after spinal cord infarction.^2^

A 95-year-old autonomous White woman with a personal history of arterial hypertension, type two diabetes mellitus, and anemia was admitted after sudden onset of motor deficits. On the previous day, the patient resorted to the emergency department with back pain with anterior bilateral irradiation and supraventricular tachycardia. After adenosine and bisoprolol administration, the patient returned to sinus rhythm with normal heart rate and was discharged. On admission, the patient presented with an asymmetric flaccid tetraparesis, proximal grade (G) 4 strength in upper limbs, G3 in elbow movement, and G2 in wrist and hand movements. In lower limbs, she had G3 in all segments on the right, and the left lower limb was plegic. Osteotendinous reflexes were only arousable at the pectoral and bicipital levels and had extensor plantar reflexes. There was no clear sensitive level and no anesthesia, but touch and pain were reduced from the abdominal segments downward. She had some proprioception errors only in the left lower limb. She was incontinent.

Blood analysis showed microcytic anemia and mild renal dysfunction. Brain computed tomography (CT) showed incipient signs of atherosclerotic microangiopathic subcortical leukoencephalopathy and discrete bilateral lenticulocapsular lacunar infarcts and nonrecent ischemic infarction in the left cerebellar hemisphere. Cervical angio-CT showed degenerative alterations, insufficient to justify the clinical presentation.

Lumbar puncture was performed: Cerebrospinal fluid analysis did not present major changes. A cervicodorsal MRI showed extensive T2-hyperintense spinal lesion from C6 until T3-T4 segments, predominantly in the central portion of the spinal cord with discrete spinal expansion and nodular, heterogeneous gadolinium enhancement. These images were suggestive of longitudinal extensive myelitis or spinal cord infarction. Transthoracic echocardiogram and 24-hour Holter showed no significant changes, and neck vessel Doppler displayed no significant stenosis. The immunological study (antinuclear antibodies; neutrophil cytoplasm antibodies; C3 and C4 complement; and IgG, IgM, and IgA) was normal, with negative anti-neuronal antibodies (anti-MOG IgG) and aquaporin-4 antibodies favoring the diagnosis of SCI. The patient underwent rehabilitation during hospital stay and began antiplatelet therapy with clopidogrel and aggressive vascular risk factor control.

According to the literature, the prevalence of SCI is 1.2% of all strokes^1^ and 5%–8% of all acute myelopathies^2^. The risk escalates in patients undergoing thoracoabdominal aortic surgery where the prevalence can be as high as 33%.^2^ The spinal cord receives its vascular support from one anterior spinal artery (ASA) and two posterior spinal arteries (PSAs) that span the length of the cord longitudinally.^2^ SCI can occur in the territory of the anterior spinal artery (ASA) or posterior spinal artery (PSA) or both.^3^ The spinal cord can be divided into three regions (medial, lateral, and dorsal), based on the distribution of each of the three arteries. There are overlapping areas among the territories of each artery.^5^ A mechanical trigger usually causes bilateral anterior or posterior spinal artery infarcts and unilateral infarcts, whereas infarcts occurring after prolonged hypotension or in arterial insufficiency are usually central and transverse infarcts.^6^

SCI may have several causes, such as aorta disease (aortic dissection, aortic thrombosis, and aortic aneurysm, among others), aortic procedures, systemic hypoperfusion, cardiogenic embolism, vasculitis, infection, hematologic disease, and spine disease, among others.^7^ One-third of ischemic strokes remain cryptogenic because diagnostic evaluation fails to reveal an exact cause.^8^

The onset of signs and symptoms caused by SCI is typically abrupt. Symptom onset to peak time usually ranges from few minutes to 48 hours.^4^ The clinical nadir is commonly reached within 1 hour of symptom onset.^9^ As the onset may be more protracted, patients with a spontaneous (i.e., nonprocedural, nontraumatic) SCI are often misdiagnosed.^3^

The most common clinical presentation is the anterior spinal artery (ASA) syndrome,^4^ which causes bilateral weakness and dissociative sensory loss with or without autonomic symptoms. On the other hand, posterior spinal artery infarction produces loss of proprioception and vibratory senses below the level of the injury and total anesthesia at the level of the injury.^1^

SCI is diagnosed in the setting of a compatible clinical syndrome without other identifiable etiology through anamnesis, examination, magnetic resonance imaging (MRI), or other examinations. If SCI is suspected, MRI is essential for diagnosis: Spinal cord swelling and “pencil-like” hyperintensities on T2-weighted images (T2WIs), enhancement on postcontrast T1-weighted images, and hyperintense signal on DWI are among the frequently encountered imaging features of SCI. Spinal cord edema will eventually be replaced by atrophy in the chronic phase. The presence of “owl's eyes pattern,” hyperintensities on axial T2WI is associated with ASA infarcts.^10^

A recent study has proposed diagnostic criteria for SCI, using the terms definite, probable, and possible SCI.^3^

The criteria included:

1. Acute nontraumatic myelopathy (no preceding progressive myelopathy)
  a. Onset to nadir severe deficits within 12 hours or less
  b. If the stuttering course is more than 12 hours, severe deficits rapidly develop within 12 hours or less
2. Magnetic resonance imaging
  a. No spinal cord compression
  b. Supportive: intramedullary T2-hyperintense spinal cord lesion
  c. Specific (one of): diffusion-weighted imaging/apparent diffusion coefficient restriction, associated vertebral body infarction, or arterial dissection/occlusion adjacent to lesion
3. Cerebrospinal fluid—noninflammatory (normal cell count, IgG index, and no oligoclonal bands)
4. Alternative diagnoses is not more likely

According to this study, our patient would be classified to have a definite spontaneous SCI because she presented with acute nontraumatic myelopathy with onset to nadir severe deficits within less than 12 hours, had an MRI with compatible changes, and there was no alternative diagnosis more likely.

Regarding the etiology, taking into account the history of supraventricular tachycardia, a cardioembolic cause is quite likely. Some studies suggest that even before atrial fibrillation or flutter develops, atrial disease may manifest as other supraventricular arrhythmias, such as paroxysmal supraventricular tachycardia (PSVT) and increased stroke risk through some combination of hypercoagulability, inflammation, endothelial injury, and structural changes.^8^ Despite these suggestive data and their potential implications, it remains unknown whether PSVT is a risk factor of stroke.^8^

Contemporary treatment concepts are adapted from guidelines for cerebral ischemia, atherosclerotic vascular disease, and acute traumatic spinal cord injury.^7^

The long-term functional prognosis is determined by the degree of spinal cord sparing.^11^ Patients with SCI have a persistently elevated mortality rate after hospital discharge. Poor prognostic factors for recovery include severe impairment at presentation, female sex, advanced age, and lack of improvement in the first 24 hours.^7^
